# Do genes and environment meet to regulate cerebrospinal fluid dynamics? Relevance for schizophrenia

**DOI:** 10.3389/fncel.2012.00031

**Published:** 2012-08-08

**Authors:** Joana A. Palha, Nadine C. Santos, Fernanda Marques, João Sousa, João Bessa, Rui Miguelote, Nuno Sousa, Paulo Belmonte-de-Abreu

**Affiliations:** ^1^Life and Health Sciences Research Institute, School of Health Sciences, University of MinhoBraga, Portugal; ^2^ICVS/3B's, PT Government Associate LaboratoryBraga/Guimarães, Portugal; ^3^Department of Obstetrics and Gynaecology, Centro Hospitalar do Alto AveGuimarães, Portugal; ^4^Universidade Federal do Rio Grande do Sul, Porto AlegreRS, Brazil; ^5^Hospital de Clínicas de Porto Alegre, Porto AlegreRS, Brazil

**Keywords:** neurodevelopment, choroid plexus, schizophrenia, cerebrospinal fluid, brain ventricles, ventriculomegaly

## Abstract

Schizophrenia is a neurodevelopment disorder in which the interplay of genes and environment contributes to disease onset and establishment. The most consistent pathological feature in schizophrenic patients is an enlargement of the brain ventricles. Yet, so far, no study has related this finding with dysfunction of the choroid plexus (CP), the epithelial cell monolayer located within the brain ventricles that is responsible for the production of most of the cerebrospinal fluid (CSF). Enlarged brain ventricles are already present at the time of disease onset (young adulthood) and, of notice, isolated mild ventriculomegaly detected *in utero* is associated with subsequent mild neurodevelopmental abnormalities similar to those observed in children at high risk of developing schizophrenia. Here we propose that altered CP/CSF dynamics during neurodevelopment may be considered a risk, causative and/or participating factor for development of schizophrenia.

## Overview

Schizophrenia is a disabling psychiatric disorder that affects 0.7–1% of the population worldwide. The disease has a heterogeneous presentation, with disorganized positive and negative symptoms that have different levels of intensity/relevance between individuals and across time in the same individual (MacDonald and Schulz, 2009). Despite the evidence for a genetic component, studies have failed to identify a disease-causative gene (Vereczkei and Mirnics, 2011). Furthermore, the lack of disease concordance in monozygotic twins strongly supports the participation of environmental factors in its etiopathogenesis (MacDonald and Schulz, 2009; Gilmore, 2010). Thus, while accepted as a neurodevelopment disease triggered by “environmental” determinants in genetic predisposed individuals, no definitive clues have emerged on which specific (combinatory) factors predispose for the disease (MacDonald and Schulz, 2009). It is, however, reasonable to propose that environmental modulators such as vitamins or hormones influence the expression of normal/mutated genes, in particular periods during (neuro)development (which in humans lasts until the second decade of life) and/or later in life, which may contribute to disease onset and establishment (Palha and Goodman, 2006; Addington and Rapoport, 2011).

Here we analyze the disease and integrate neurodevelopment, genes and environment, from a novel perspective, bringing into play the most well-characterized and consistent pathological feature in schizophrenia: the enlargement of the brain ventricles (Steen et al., 2006). Whether brain ventricle enlargement is secondary to atrophy of the surrounding brain parenchyma, namely the cortical formation, or to an increased intracranial pressure, due to altered dynamics of the cerebrospinal fluid (CSF) (production, excretion), has never been considered and/or addressed. In fact, the literature recognizes that the meaning and the origin of the enlarged ventricles in the schizophrenic brain is still not known (Steen et al., 2006; Vita et al., 2006). The brain ventricles are filled with CSF, a fluid mostly produced by the choroid plexus (CP), a monolayer row of epithelial cells originating in the ependymal cell layer that lines the brain ventricles. In the adult human brain, approximately 160 mL of CSF fill the ventricles, subarachnoid space, and spinal cord, which are renewed about three to four times per day, highlighting the secretory capacity of the CP. This capacity, higher than any other epithelia (Brown et al., 2004), may relate to the requirement of a tight regulation to maintain brain homeostasis. By modulating the composition of the CSF, the CP influences the delivery of nutrients, growth factors, and other molecules to the brain; in addition, it also contributes to the excretion of metabolites out of the brain (Johanson et al., 2008). Furthermore, the CP is highly irrigated and therefore able to rapidly respond to peripheral stimuli, as we recently showed to be the case in response to acute peripheral inflammation (Marques et al., 2009b), adding to several studies, by our and other laboratories, demonstrating that changes in the CP transcriptome result in altered concentration of specific receptors and transporters, as well as in the altered secretion of hormones and carrier proteins into the CSF (Emerich et al., 2005; Marques et al., 2008, 2009c). These alterations have been implicated in neurodegenerative disorders such as Alzheimer's disease and multiple sclerosis (Reboldi et al., 2009; Vargas et al., 2010). A further interesting finding, not extensively reported in the literature, is the relatively high incidence of calcifications in the CP of schizophrenic patients, which may, as well, result from altered CP metabolism (Belmonte de Abreu, unpublished data and Bersani et al., 1999).

Here we theorize that, in the schizophrenic brain, events during (neuro)development may interfere with the CP's activity or with the ability of the brain to circulate the CSF, and that this may relate with schizophrenia etiology and pathology, including ventricle enlargement. With this article we intend to bring into the research field the possible role of the CP–CSF nexus in predisposing for/modulating schizophrenia.

## The ventricular triad: brain ventricles, choroid plexus, and cerebrospinal fluid during development

The CP is a structure highly conserved both phylogenetically and ontogenetically (Johansson et al., 2008). During ontogenetic development, the ventricular lining is constituted by proliferating cells, which generate all types of neuroectodermal brain cells, the ependymoglial cells among which the ependymal cells and CP epithelial cells are the most prominent ones (Dziegielewska et al., 2001). At this stage the CP is, relatively to the brain parenchyma, much bigger than in adulthood. The same applies to the lateral ventricles, whose diameter remains stable (mean 6.4 ± 1.2 mm) from 15 weeks of gestation until the term of pregnancy (Salomon et al., 2007). In the adult, the CP is positioned within the ventricles of the brain: one in each lateral, one in the third, and one in the fourth ventricle. Grossly, the CP is lobulated with a unique and continuous line of epithelial cells originating from the ependymal line of the ventricles and it “floats” in the CSF space within the ventricles. The apical side of the epithelial cells faces the CSF while the basolateral side faces the blood, lying, therefore, in the stroma side in contact with a large number of fenestrated capillaries. Of note, at the levels of the CP the capillaries are fenestrated, meaning that there is no blood-brain barrier. Therefore, within the ventricles, the CP epithelial cells constitute the barrier that separates the blood from the CSF and brain parenchyma. In fact, early during embryogenesis, within the first weeks of gestation, the CP already constitutes a fundamental functional barrier (Dziegielewska et al., 2001).

Due to its localization, the CP is ideally positioned to delivery molecules and nutrients into and out of the brain. For that, it is well equipped with transporters and receptors, both in the apical and basolateral membranes and is also a prominent source of neuropeptides, growth factors, and cytokines in the CNS during development and in adulthood (Bondy et al., 1990; Yamamoto et al., 1996; Strazielle and Ghersi-Egea, 2000; Krizhanovsky and Ben-Arie, 2006). During development the CP displays a recognized paracrine function essential for cerebellum formation, with the effects mediated through temporally regulated retinoic acid production (Yamamoto et al., 1996, 1998). Similarly, for example, the insulin growth factor 2, a morphogen also secreted by the CP into the CSF, stimulates neural cortical progenitors (Lehtinen and Walsh, 2011).

The relevance of embryonic CSF-born molecules to brain development is even greater if we consider that apical progenitors (neuroepithelial cells and radial glial progenitors) lie adjacent to the ventricular surface and are bathed by the CSF. In fact, the influence of the CSF content over neural progenitor cells is not restricted to the embryo. In adult mice it was shown that the pattern of CSF flow inside the brain ventricles influences the pattern of subventricular zone (SVZ) neuroblasts migration toward the olfactory bulbs; a process known to be regulated by CP secreted proteins such as Slit 1 (Sawamoto et al., 2006). Finally, it is of note that several proteins known to modulate neurogenesis are induced in the CP in response to an inflammatory stimulus/infection (Marques et al., 2009a,b,c). This is of interest if one considers that inflammation/infection during embryonic development has been associated with increased risk of schizophrenia (Brown, 2011; Meyer, 2011). Independently from the cause of altered neurogenesis, and in the context of the present discussion, it is of interest to consider whether a faulty neurogenesis during development may also be at the root of the morphological and/or neurological findings in the schizophrenic brain (including ventricle enlargement), or whether faulty neurogenesis would provide increased sensitivity to further neurodegeneration at maturity.

A recent study in cynomolgus monkey fetuses suggested that, during normal development, the morphological maturation of the lateral ventricle is linked to cortical maturation. During the fetal period the decrease in ventricular volume is accompanied by a decrease in the volume of the germinal matrix around lateral ventricles and an increase in the depth of the calcarine sulcus (Fukunishi et al., 2011). A study using magnetic resonance imaging (MRI) of postmortem human fetuses showed that the reduction of the lateral ventricle volume after 23 weeks of gestation is paralleled by a decrease in volume of the germinal matrix, in contrast to the expansion of the subcortical structures (Kinoshita et al., 2001). Alterations in the ventricular maturation may, therefore, interfere with the proper development of other brain regions. In accordance, a prospective study from our group in human fetuses with isolated mild ventriculomegaly and controls, showed an inverse association between depth of the calcarine and parieto-occipital fissures (a marker of cortical maturation) and the ventricular diameter. The depth of these fissures was smaller in fetuses with ventriculomegaly than in controls (Miguelote, unpublished data). Most cases with asymmetric ventricles also displayed asymmetry of the fissures, with the fissure depth lower in the affected side. This association decreases at later gestational ages, which may mean that the difference observed at early gestational ages is caused by a delay in the cortical maturation. Abnormalities in ventricular and cortical maturation are pertinent in light of other findings observed in postmortem brain studies of schizophrenic patients, such as the reduced dendritic spine density in pyramidal neurons in input layers of the prefrontal cortex, and abnormally oriented, with signs of arrested migration, hippocampal neurons (Glantz and Lewis, 2000; MacDonald and Schulz, 2009).

Altogether, enlarged lateral ventricles during development may ultimately be the result of altered CSF formation, circulation, and/or homeostasis. We will next briefly address developmental ventriculomegaly.

## Ventriculomegaly

Ventriculomegaly, defined as an atrial diameter ≥ 10 mm, is a frequent finding on prenatal ultrasound examination (1% of the fetuses). It can be associated with chromosomal abnormalities, congenital infection, cerebral vascular accidents or hemorrhage, and other fetal cerebral and extracerebral abnormalities. The possible mechanisms causing fetal ventriculomegaly are obstruction to CSF flow, hypersecretion of CSF (rare), defective filtration of CSF (rare), and altered development of the intracranial architecture. On this, aqueductal stenosis is the most common reason of obstruction to CSF flow and can be caused by infections, bleeding, or other pathologies that cause gliosis and obliterate the aqueduct (D'Addario et al., 2007). Sometimes a few rudimentary ependymal-lined tubules are seen in place of the aqueduct (aqueductal forking). A multifactorial pattern of inheritance is probably the cause, but in 5% of all cases an X-linked transmission is present (D'Addario et al., 2007). The most frequent fetal cerebral abnormalities presented in fetus with ventriculomegaly are agenesis of the corpus callosum, agenesis, or hypoplasia of the cerebellar vermis (Dandy–Walker malformation), spinal neural tube defect (Chiari II malformation), and cortical migrational abnormalities. Even when the ventriculomegaly is mild (atrial diameter between 10 and 15 mm) the fetuses are still reported to present an increased risk of abnormal or delayed neurodevelopment in infancy (about 11%) (Melchiorre et al., 2009). The progression of ventricular dilatation is one of most important prognostic factors; progression occurs in about 15% of cases, regression in about 32% and the remaining stay stable (Kelly et al., 2001).

Prenatal isolated mild ventriculomegaly has been suggested to be associated with neuropsychiatric disorders, including autism, attention deficit hyperactivity disorder, learning disabilities, and schizophrenia by several case series (Piven et al., 1995; Gilmore et al., 1998, 2001). Nonetheless, long-term longitudinal series are needed and should strongly be considered, particularly in light of the findings relating the CP/CSF with ventricle volumes in the schizophrenic brain as we will next address.

## Brain ventricles and the CP/CSF nexus in the schizophrenic brain

Throughout the last decade, multiple systematic and separate meta-analysis reviews of MRI studies, which report on quantitative measurements of volumes of brain regions in schizophrenic patients (in first-episodes and on longitudinal assessments; compared to healthy controls), concluded that significant overall effect sizes were consistently demonstrated for total (particularly, lateral and third) ventricular volume increase (Elkis et al., 1995; Wright et al., 2000; Steen et al., 2006; Vita et al., 2006; Kempton et al., 2010; Olabi et al., 2011). While ventricle enlargement is the most robust pathological finding in schizophrenia (Shenton et al., 2001), the precise moment of ventricle enlargement is not known. Nevertheless, current evidence proposes a two-hit model, with size and shape differences already present before illness onset (Rosa et al., 2010; Andreasen et al., 2011) and progressively increasing during the course of illness (at an annual rate of +0.36% for lateral ventricle volume) (Olabi et al., 2011). Of interest, several studies report that the ventricle enlargement is suggested to be a disease-specific event since no differences are found in ventricular volume when non-affected siblings are compared to control individuals, while there are marked differences between patients and relatives or the general population (Goldman et al., 2008; Boos et al., 2011). On this, it should be noted that, beyond schizophrenia, enlargement of the lateral ventricles can be associated with other neuropsychiatric disorders, such as bipolar disease and major depressive disorder (Kempton et al., 2008, 2011). Still, on schizophrenia, a genetic component of ventricular enlargement was suggested from studies in discordant twins when compared to mentally healthy twin pairs (Brans et al., 2008). These observations indicate that while progressive brain volume changes seem at least partially mediated by unique environmental factors, significant additive genetic influences should be considered on the correlation between volume change during development and course of schizophrenia phenotype.

An additional aspect to be considered relates to the location in the volume changes. Three-dimensional MRI (Meduri et al., 2010) revealed volume differences of 26%, more pronounced in the occipital horn (41% left, 39% right), but also present in the frontal horn (20.5% left, 23% right), main body (19% left, 21% right), and temporal horn (−0.7% left, 13% right) (Yotsutsuji et al., 2003). These may reflect the tension and plasticity of white and gray matter and CSF, occurring at different levels of lateral and third ventricles. Cortical CSF volume increases in the bilateral prefrontal, temporal, and right orbital areas have been associated with illness duration (Molina et al., 2002). These observations support that neurodegenerative events occur throughout the course of the disease. This is particularly interesting if one considers the current view that schizophrenia originates from more than a single event. The hypothesis proposed here suggests alterations in the CP/CSF nexus as a possible key element in such succession of events.

These observations should now bring us to the reasons of the observed ventriculomegaly. Ventricular enlargement can result either from brain parenchyma atrophy, leading to a novel equilibrium by filing the space with CSF; or enlarged ventricles result from increased CSF production or/and decreased CSF excretion, during specific times in development, that force the brain parenchyma to “shrink” or to allow the ventricles to occupy a larger space. Notably, in a naturally occurring rat model of early onset (E18) hydrocephalus, the obstruction of natural CSF flow results in abnormal cell proliferation and decreased number of cells migrating (Mashayekhi et al., 2002). Furthermore, *in vitro* studies demonstrated that CSF from these hydrocephalic rats arrested cell division in the S-phase (Owen-Lynch et al., 2003). In this perspective, increased CSF pressure may not only physically compromise the brain parenchyma, but also possibly, through an altered secretome, modulate neural development in such a way that the developing brain becomes more susceptible to a second triggering key event in disease etiology.

Among these, a possible target of environmental triggering events in the susceptible brain is the ongoing neurogenesis in the adult brain [first described in the 60's by Altman (1962)]. The birth, survival, and differentiation of new neurons in the adult brain have been identified in two distinct areas: the subgranular zone (SGZ) of the hippocampal dentate gyrus (DG), and the SVZ. In the SGZ, the neural precursor cells generate a large pool of neuroblasts and immature neurons that undergo a selection process that ends in the survival and integration of a small number of mature and functional neurons in the adjacent granular layer of the DG. In the SVZ, neural precursor cells reside in the walls of the lateral ventricles and divide to give rise to neuroblasts that migrate along the narrow rostral migratory stream (RMS) to the olfactory bulb. While several studies have suggested a role of adult neurogenesis in distinct cognitive domains (Shors et al., 2002), the functional importance of this process remains unclear. Importantly, it is a highly regulated process by the action of neurotransmitters, growth factors, and hormones. In particular, stress-induced glucocorticoid secretion downregulates adult neurogenesis in the SGZ, while antidepressant treatment in rats increases cell proliferation in this area (Malberg et al., 2000). These observations have led to conflicting results on the role of hippocampal newborn neurons in depression and in the action of antidepressant drugs (Santarelli et al., 2003; Bessa et al., 2009). Nonetheless, the analysis of hippocampal neural stem cell proliferation in the postmortem brains of patients with depression, bipolar disorder, and schizophrenia revealed a significant decrease of proliferation in the DG in the schizophrenic brains (Reif et al., 2006). Furthermore, preclinical studies in rodents have revealed that adult neurogenesis ablation, by irradiation of the hippocampus and SVZ, leads to behavioral deficits associated to schizophrenia (Iwata et al., 2008). Also, chronic treatment with antipsychotic drugs can potentiate adult neurogenesis in the hippocampus and in the SVZ. Interestingly, first-generation antipsychotics like haloperidol stimulate neurogenesis in the SVZ while the effects of second-generation antipsychotics like clozapine are mainly observed in the SGZ of the hippocampus (Newton and Duman, 2007). By modulating the CSF composition, alterations in the CP transcriptome in the adult may, independently from the ventricle size, influence adult neurogenesis, again bringing the CP/CSF nexus into the disease.

Based on this scattered but coherent evidence, we believe that a novel provocative view on how the CP/CSF nexus relate to enlarged brain ventricles/CP/CSF, and other features observed in schizophrenia, may be proposed. Testing the hypothesis is certainly challenging, when considering the limitations of studies of schizophrenia symptoms and features in animal models on one hand and the requirement of long longitudinal human studies on the other. In any case, we next address how to investigate this hypothesis, both in humans and in animal models of the disease.

## How to test the hypothesis?

### Animal studies

The establishment of a working animal model of schizophrenia is a particular challenge given that no unifying model is available (Jaaro-Peled et al., 2010; Nestler and Hyman, 2010); however, various animal models display neurological signs of the disease, which can be measured. In fact, for phenotype assessment, it is well accepted that prepulse inhibition (PPI) deficit is indicative of disrupted sensorimotor gating, a cognitive process that prevents sensory overload, and the key indicator for a schizophrenia-like phenotype in animal models of the disease (Powell et al., 2009; Nestler and Hyman, 2010). On established models of schizophrenia (including neurodevelopment and/or ventriculomegaly), characterization of the CP transcriptome and secretome could provide clear indication on potential molecular pathways related to the disease. These may be of various natures: secretory pathways that will result in altered CSF composition, ultimately influencing the brain's interstitial fluid and neuronal/glial function; transporters that may modulate the access of specific blood-born molecules including nutrients, growth factors, cytokines, or metabolite excretion out of the brain, again with impact on the CSF composition; and receptors, both in the basolateral and apical membranes of the CP epithelial cells, which may participate in modulation of the secretory machinery [as we recently showed to be the case with respect to inflammation and iron metabolism (Marques et al., 2009a,b,c)]. Next we will briefly describe some of the models that might prove to be particularly of interest to study from a CP/CSF nexus perspective in the context of schizophrenia, including: four animal models induced during pregnancy (food deprivation, stress, administration of inflammatory mediators, disruption of neurogenesis), one induced in the neonatal period (excitotoxic lesions in the ventral hippocampus), the disrupted-in-schizophrenia (DISC1) null mouse model that displays ventriculomegaly, and the spontaneously hypertensive rat that presents ventriculomegaly as a result of increased CSF secretion.

The prenatal models (Meyer and Feldon, 2010) are thought to be relevant from human epidemiological studies showing that the risk of developing schizophrenia is higher upon prenatal (or perinatal) insults such as maternal exposure to malnutrition, stress, infection, and/or immune activation (as reviewed: Tandon et al., 2008a,b; MacDonald and Schulz, 2009). With respect to malnutrition/prenatal famine (the consequences of which, in schizophrenia incidence, have been derived from epidemiological studies such as the “Dutch Hunger Winter” and the “Chinese Famine,” and others, as reviewed by Brown and Susser, 2008), the condition is replicated in animal models by prenatal protein deprivation of pregnant rats (reviewed, Meyer and Feldon, 2010) so to explore, in the offspring, alterations in dopamine neurochemistry and deficits in PPI, prominent characteristics of the disease (Toda and Abi-Dargham, 2007). Other prenatal risk factors can also be mimicked: treatment of pregnant rats with dexamethasone during the final week of gestation constitutes a unifying model of stress involvement in schizophrenia (Koenig et al., 2002), while lipopolysaccharide (bacterial endotoxin) administration of rats at later stages of pregnancy, evidences that environment (infection) risk factors during pregnancy may increase the incidence of schizophrenia, as measured by deficits in sensorimotor gating (Fortier et al., 2007). Finally, administration of methylazoxymethanol acetate to pregnant dams, a model that explores the disruption of neurogenesis during neurodevelopment in the offspring (Talamini et al., 1998; Moore et al., 2006), is also a consistently used prenatal model of schizophrenia. Altogether, these models also fulfill the expectation of the neurodevelopmental theory of schizophrenia, as pointed out by Meyer and Feldon (2010). However, other “non-exposure” models are also relevant from a neurodevelopmental perspective. At the neonatal period, excitotoxic lesion(s) in the ventral hippocampus model are used to simulate neurobiological aspects of schizophrenia; interestingly, after puberty these animals present a schizophrenia-like behavioral phenotype (Tseng et al., 2009). The spontaneous hypertensive rat, which displays both ventriculomegaly (Ruchoux et al., 1992) and signs of altered dopaminergic signaling (van den Buuse, 2004), could also be an especially relevant animal model to scrutinize in the context of the CP–CSF nexus and ventricular enlargement in schizophrenia. Finally, from a genetic perspective, the *Disc1* model of schizophrenia is of particularly interest. Mutant *Disc1* mice present enlarged lateral ventricles in juveniles, and behavioral aspects similar to the pathology of patients with schizophrenia (Pletnikov et al., 2008; Ayhan et al., 2011; Brandon and Sawa, 2011).

The *Disc1* gene encodes for DISC1, a scaffold protein widely expressed throughout the fetal and adult brain (albeit particularly prominent in the human hippocampus), which contains domains for proteins involved in neurodevelopment, cytoskeletal function, neurite outgrowth, and cAMP signaling (Wexler and Geschwind, 2011). Common DISC1 polymorphisms are associated with neuropsychiatric phenotypes including altered cognition, and brain structure and function (Brandon et al., 2009; Ayhan et al., 2011; Brandon and Sawa, 2011). How DISC1 regulates these different aspects is not well understood; however, recent studies have greatly elucidated on possible mechanisms and provided clues of DISC1's plethora of actions. For example, on neuronal development, Kang et al. (2011) have shown that DISC1 interacts with fasciculation and elongation protein zeta-1 (FEZ1)to synergistically regulate dendritic growth of newborn neurons in the adult mouse hippocampus. Genetic association studies in cohorts of schizophrenia patients further revealed an epistatic interaction between FEZ1 and DISC1 for risk of schizophrenia. Wang et al. (2011) explored DISC1 in synaptic function, reporting Nck-interacting kinase (TNIK) as a key synaptic partner for DISC1, providing evidence that the DISC1–TNIK interaction regulates synaptic composition and activity by stabilizing the levels of key postsynaptic density proteins. Finally, also recently, common DISC1 polymorphisms have been identified to disrupt Wnt/GSK3β signaling and, consequently, brain (neuro)development (Singh et al., 2011).

While information obtained from animal data may provide proof of concept for the proposed hypothesis, this will always be limited given the impossibility to directly translate the observation into humans. Nonetheless, studying the CP transcriptome in these various models will certainly bring into the equation-specific pathways that may be common among all and offer key-insights for human studies considerations.

### Human studies

Addressing how the CP/CSF nexus may be involved in the etiology of schizophrenia will certainly be a difficult endeavor to study in humans. It is one of the aims of this article to challenge others in the scientific community to either revisit medical records or information from schizophrenic patients, or to encourage the design of longitudinal studies that may contribute to test this hypothesis.

In human studies, a feasible way to get insight on the CP's possible involvement in schizophrenia could be by checking whether genes that emerge from the characterization of the CP transcriptome in the animal models have been linked or associated with the disease. Of interest, aquaporins 1, 4, and 10, which are abundant in the CP, are within the genetic *loci* mostly implicated in schizophrenia (7p15.1, 18q11.2, 1q21.3). We have recently characterized the CP mouse transcriptome in basal conditions (Marques et al., 2011) and could next search, in the publically available databases, whether CP genes have been identified as altered in brains from schizophrenic patients or have been associated with the disease. While a positive association study may be encouraging, we are aware that a negative finding on the transcriptome studies does not exclude the hypothesis, since no study to data has specifically addressed the gene expression profile in the CP tissue of patients. Studying the CP transcriptome from patients would certainly be another approach. Again, and as for any other transcriptome study in patients suffering from a chronic disease and subjected to various and prolonged medication, a definitive link between the transcriptome and the disease cannot be fully established given the influence of the disease in itself and of the treatment, on top of other medical conditions that may have developed in the meantime.

A more valuable but less feasible (but not impossible!) approach would be to follow newborns, who have been identified with mild ventricle enlargement on fetal ultrasound scan, into adulthood, with a detailed characterization of behavior. The lateral ventricle width is routinely assessed on second trimester ultrasound examination. Furthermore, ventriculomegaly is a relatively common finding (≅1%) of clinical relevance as a marker of a variety of underlying disorders (chromosomal abnormalities, congenital infection, cerebral vascular accidents or hemorrhage, and other fetal cerebral and extracerebral abnormalities). When ventricle dilatation is mild (10–15 mm), and there are no associated anomalies, the risk of abnormal or delayed neurodevelopment in infancy is still higher than the estimated for the general population. The association between prenatal isolated mild ventriculomegaly and neuropsychiatric disorders remains, however, to be established.

A final suggestion would be to monitor ventricular enlargement in the progeny of patients with schizophrenia, as an attempt to identify how early ventricular enlargement is observed in individuals that ultimately develop the disease; and to follow first episode patients on how additional ventricular enlargement may relate with disease progression and response to treatment. It will always be difficult to discriminate between ventricular volume findings and other genetic aspects that may predispose the progeny of schizophrenic patients for disease development, but this will certainly be a piece of information to add to the others proposed this far.

## Concluding remarks

**Figure 1:**
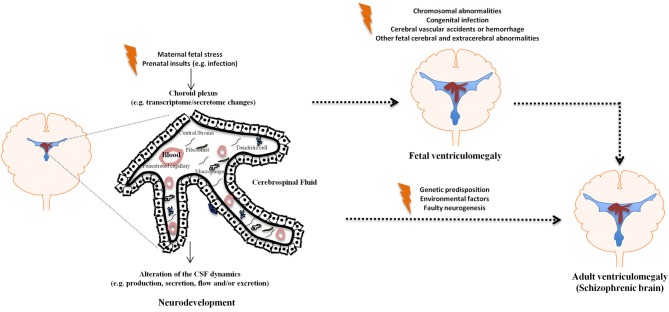
**The choroid plexus/CSF nexus in ventriculomegaly.** The figure depicts schematically how various insults may influence the CP/CSF homeostasis during development and in the adult brain, resulting in ventriculomegaly.

The brain barriers have only recently started to be seen as more than mere obstacles for the passage of molecules and cells in and out of the brain. It is now clear that they actively participate in modulating brain homeostasis in health and in disease (Marques et al., 2009a,b; Zlokovic, 2010). Despite the fact that the CP is on the interface between the CNS and the periphery, and thus in a privileged position to mediate interactions between the periphery and the CNS, little attention has been devoted to the CP in psychiatric disorders. Regarding schizophrenia in particular, several lines of evidence point to the CP/CSF nexus as a potential site of altered homeostasis; chiefly among which, enlarged ventricles are the common pathological finding in the schizophrenic brain, and no data is available on whether this is simply a consequence or a causative event in the disease. Surprisingly, both aspects, the CP privileged location and functions, together with the enlarged ventricles findings, have remained vastly unexplored in schizophrenia research. Nonetheless, it is reasonable to expect that maternal condition such as exposure to pathogens/infection, malnutrition, or stress, all implicated in schizophrenia, influence: (1) the CP secretory rate, as well as its secretory/homeostatic profile, ultimately resulting in enlarged ventricles and (2) neurogenesis/cortical migration. In summary, as depicted in Figure 1, both during development and in the adult brain, it is reasonable to suggest that the CP/CSF nexus may influence the onset and progression of schizophrenia.

### Conflict of interest statement

The authors declare that the research was conducted in the absence of any commercial or financial relationships that could be construed as a potential conflict of interest.
